# Mode of birth in monochorionic versus dichorionic twin pregnancies: a retrospective study from a large tertiary centre in Germany

**DOI:** 10.1186/s12884-022-04531-3

**Published:** 2022-03-17

**Authors:** Lena Wandel, Harald Abele, Jan Pauluschke-Fröhlich, Karl Oliver Kagan, Sara Brucker, Katharina Rall

**Affiliations:** Department for Women’s Health, Women’s University Hospital, Calwerstraße 7, 72076 Tübingen, Germany

**Keywords:** Twin pregnancy, Dichorionic-diamniotic twins, Monochorionic-diamniotic twins, Mode of delivery, Vaginal delivery

## Abstract

**Background:**

Optimal mode of birth for twins, in particular monochorionic twins, has been the subject of much debate. This retrospective study compared maternal and newborn outcomes after vaginal birth in monochorionic and dichorionic twins, utilizing a large institutional database.

**Methods:**

Retrospective analysis focusing on 98 monochorionic-diamniotic (MC-DA) and 540 dichorionic-diamniotic (DC-DA) twin births extracted from the perinatal database of a large German hospital. Pregnancies ≥36 weeks of gestation with two viable foetuses born between 2004 and 2014 divided into planned vaginal and planned caesarean delivery were included. Descriptive analysis was performed for maternal characteristics. Odds ratios (OR) with 95% confidences intervals (CI) tested the predictive effect of vaginal birth on neonatal and maternal outcomes.

**Results:**

51.0% MC-DA and 46.7% DC-DA twin pregnancies were planned vaginal births and 44.0% MC-DA mothers and 43.7% DC-DA mothers actually gave birth vaginally. The overall rate of caesarean section (CS) during the years under observation was 79.6% for MC-DA and 77.0% for DC-DA pregnancies. There were no significant differences in neonatal outcome between the subsamples, although acidosis was observed more often in the second DC-DA twin and Apgar scores < 7 were observed more often in MC-DA twins.

**Conclusion:**

Vaginal birth may be recommended as an option to women with monochorionic twins as no significant differences in outcomes were found between MC-DA and DC-DA twins. However, over half of planned vaginal twin births resulted in CS.

## Background/introduction

The incidence of multiple births has risen over the last years. In Germany, the proportion of multiple births has more than doubled over the last 40 years and in 2019, 1.84% of newborns were multiples. This trend can also be seen in other European countries such as England, Wales and France [1–3]. Reasons cited for this increase include advanced age of women at the time of conception and the extended use of assisted reproductive technology, such as in-vitro-fertilization, intracytoplasmic sperm injection and ovarian stimulation [3–5]. Multiple pregnancies are associated with higher maternal and neonatal risks. Mothers of multiples are more often affected by obstetrical complications and surgical interventions [2, 3, 6]. Chorionicity is responsible for adverse perinatal outcomes for twins [3, 7, 8]. About half of all twins are born before 37 weeks’ gestation or with a birth weight < 2500 g [9]. Monochorionic twins have higher rates of mortality and morbidity than dichorionic twins and peripartal mortality is approximately twice as high than for dichorionic twins [4, 7, 10, 11]. There has therefore been much discussion in the past about the optimal mode of birth for multiple pregnancies. Some studies have shown a higher risk of adverse outcomes for both twins or the second twin after vaginal birth compared to planned CS, which led to a large increase in CS rates during the last years [9, 12–16]. The findings of the Twin Birth Trial were ambivalent with regard to vaginal versus caesarean birth for twin pregnancies: the study showed neither a significant increase or decrease in the risk of foetal or neonatal death or morbidity for twin pregnancies between 32 + 0 and 38 + 6 weeks of gestation, with the lead twin in cephalic position [17]. Other studies have also shown that vaginal delivery is an option for twins regardless of chorionicity when taking specific criteria into account [18–20]. The objective of this study was to investigate the neonatal outcomes and maternal complication rates after vaginal birth of monochorionic twins compared to dichorionic twins at a single institution.

## Methods

This retrospective cohort study covers a period of 11 years (2004–2014) and includes twin births at the Department of Women’s Health, University Hospital of Tuebingen (UFK), Germany. The UFK is a level one perinatal centre with over 3500 births per year and a large percentage of multiple births. Data was extracted from an extensive birth registry (i.s.h. med, SAP for Healthcare, Cerner, North Kansas City, MO, USA). In the 11 years under observation, 1120 twin births were recorded. All twin births ≥36 weeks’ gestation with two viable foetuses were included in the analysis. Of the 638 twin births meeting these criteria, subsets for comparison were created between MC-DA and DC-DA twin pregnancies with planned vaginal birth, according to an intention-to-treat analysis. Successful planned vaginal birth was defined as including instrumental births and a combination of vaginal and instrumental birth. Crosschecking for plausibility was performed prior to analysis. Neonatal outcomes included 5-min Apgar scores, pH-values, need for breathing support (intubation and CPAP), birthweight and mortality, all separated for the first and second twin. Admission to neonatal intensive care unit (NICU) was defined as need for breathing support. Saling’s classification for pH-values was applied, pH-values < 7.20 were assigned to acidosis and pH-values between 7.20 and 7.24 to preacidosis [21]. Adverse neonatal outcomes included acidosis, five-minute Apgar < seven and admission to NICU. Maternal outcome was defined as postpartum blood loss.

### Ethics approval

The study was approved by the local ethics committee (Ethics Committee, Department of Medicine, Eberhard Karls University and University Hospital Tuebingen, Germany; 398/2019BO2).

### Statistical analysis

SPSS (IBM Corp. Released 2019. IBM SPSS Statistics for Windows, Version 26.0. Armonk, NY: IBM Corp), Microsoft Office Excel (Version 2016) and LaTex were used to analyse the data and to create figures, tables and graphs. Evaluation of normal distribution of variables was performed using Kolmogorov-Smirnov- and Shapiro-Wilk-tests. Medians and ranges were estimated based on non-normally distributed data. Cross tables and percentages for categorial data were performed and Chi-square-test or Fisher’s-exact-test were used to compare categorial data. The decision was made to forego multivariant testing for the subsamples as none of the variables was found to be significant. The Mann-Whitney-U-test was applied for non-normal distribution. Odds Ratio and 95% confidence intervals were calculated for adverse neonatal outcomes comparing vaginally born monochorionic versus dichorionic twins. *P*-values for hypothesis tests were two-sided and a *p*-value < 0.05 was considered statistically significant.

## Results

Between 2004 and 2014 there were 1120 twin births at UFK, 98 of which were MC-DA and 540 DC-DA and fitted the inclusion criteria. One monochorionic-monoamniotic twin pregnancy ≥36 weeks of gestation was excluded, as monochorionic-monoamniotic twins are always elective CS according to hospital protocol. In total, 302 (47.3%) were planned vaginal births and 336 (52.7%) were planned CS births. Vaginal birth was achieved for 132 pregnancies (43.7%), whereas 155 births (51.3%) ended in CS. Additionally, 15 twin pairs (5.0%) were born with a combination of vaginal and caesarean delivery. The mode of birth for the entire cohort was 77.0% CS, 20.7% vaginal and 2.3% combination vaginal and CS.

**Fig. 1:**
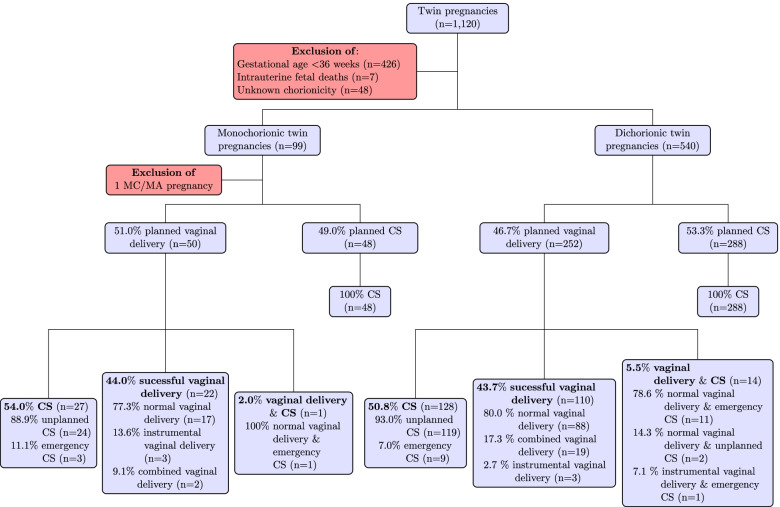
Study collective distributed into planned and actual mode of delivery 2004–2014 at UFK Tuebingen

Of the 51.0% of MC-DA pregnancies with planned vaginal births, 44.0% achieved this goal. 77.3% had a normal vaginal birth, 13.6% a vaginal instrumental birth and 9.1% were delivered by a combination of vaginal and instrumental birth (Fig. 1). Planned MC-DA vaginal birth resulted in CS for 54.0% of women and 2.0% delivered one twin as a vaginally and the second with CS.

For the 46.7% of women with a DC-DA twin pregnancy and a planned vaginal delivery, 43.7% were able to achieve their goal, while 50.8% experienced a CS and 5.5% a combination of vaginal and caesarean birth (Fig. 1).

In the 11 years under observation, MC-DA twin pregnancies had a CS rate of 76.5% and vaginal birth rate of 22.4%. For women with a DC-DA twin pregnancy, the rates were 77.0% for CS and 20.4% for vaginal births. Combined vaginal and caesarean births accounted for 1.0 and 2.6% of MC-DA and DC-DA twin pregnancies, respectively (Fig. 1).

### Comparison according to chorionicity

Differences in the demographic characteristics of women with MC-DA and DC-DA pregnancies and planned vaginal births did not reach statistical significance (Table 1). Women in the MC-DA twin cohort were in median 2 years younger than those in the DC-DA group (31.0 and 33.0 years respectively). Pre-pregnancy body mass index (BMI) and BMI at birth were lower in the MC-DA subsample with successful vaginal birth than in the DC-DA group (Table 2). Gestational age for both groups was 38 weeks.

**Table 1:** Baseline characteristics and distribution of deliveries during the study period

	Planned vaginal delivery (*n* = 302)		
	MC/DA (*n* = 50)	DC/DA (*n* = 252)	*p*
Age	31.0 (19–40)	33.0 (20–44)	0.266
Parity	0 (0–3)	0 (0–5)	0.969
BMI before pregnancy^a^ (kg/m^2^)	23.30 (15.9–34.0)	23.05 (16.4–46.3)	0.500
BMI before delivery^b^ (kg/m^2^)	28.73 (21.6–40.7)	29.37 (20.7–55.6)	0.332
Gestational age at delivery (weeks)	37 (36–41)	37 (36–41)	0.231
Mode of delivery (%)			
Caesarean	27 (54.0)	128 (50.8)	–
Planned CS	-	-	
Unplanned CS	24 (88.9)	119 (93.0)	
Emergency CS	3 (11.1)	9 (7.0)	
Vaginal	22 (44)	110 (43.7)	
Normal vaginal	17 (77.3)	88 (80.0)	
Instrumented vaginal	3 (13.6)	3 (2.7)	
Combined vaginal	2 (9.1)	19 (17.3)	
Combined vaginal & CS	1 (2.0)	14 (5.5)	

Medians, ranges and percentages were estimated

^a^ 13 (planned vaginal, DC/DA) and 2 (planned vaginal, MC/DA) unknown cases which are not taken into account

^b^ 15 (planned vaginal, DC/DA) and 2 (planned vaginal, MC/DA) unknown cases which are not taken into account

**Table 2:** Characteristics of vaginal delivery divided into MC-DA and DC-DA pregnancies

		Vaginal delivery (*n* = 132)		
	MC/DA (*n* = 22)	DC/DA (*n* = 110)	Odds Ratio (95% CI)	*p*
Age	31.5 (22–40)	32.0 (21–43)		0.905
Parity	1 (0–2)	1 (0–4)		0.415
BMI before pregnancy^a^ (kg/m^2^)	22.74 (19.1–34.0)	22.94 (18.3–46.3)		0.940
BMI before delivery^b^ (kg/m^2^)	28.68 (23.8–40.7)	29.30 (22.6–46.6)		0.873
Gestational age at delivery (weeks)	38 (36–41)	38 (36–41)		0.885
pH				
1st twin^c^	7.26 (7.12–7.41)	7.29 (7.03–7.40)		0.502
2nd twin^c^	7.27 (7.09–7.35)	7.23 (7.02–7.36)		0.105
Acidosis (%)				
1st twin^c^	3 (13.6)	11 (10.2)	1.392 (0.355–5.469)	0.705
2nd twin^c^	3 (13.6)	37 (34.3)	0.303 (0.084–1.091)	0.056
any twin^f^	5 (22.7)	41 (38.3)	0.473 (0.162–1.381)	0.164
Apgar 5′				
1st twin	9 (4–10)	9 (2–10)		0.654
2nd twin	9 (3–10)	9 (6–10)		0.171
Apgar < 7 5′(%)				
1st twin	1 (4.5)	1 (0.9)	5.190 (0.312–86.290)	0.307
2nd twin	1 (4.5)	2 (1.8)	2.571 (0.223–29.666)	0.424
any twin	1 (4.5)	3 (2.7)	1.698 (0.168–17.128)	0.522
Birth weight (g)				
1st twin	2605 (2120-3350)	2625 (1890-3640)		0.903
2nd twin	2570 (1878-3270)	2605 (1900-3730)		0.330
Admission to NICU (%)				
1st twin^d^	1 (4.8)	2 (1.8)	2.700 (0.234–31.208)	0.411
2nd twin^e^	2 (9.5)	8 (7.3)	1.329 (0.262–6.750)	0.664
any twin^e^	3 (14.3)	10 (9.2)	1.650 (0.413–6.588)	0.440
Intubation (%)				
1st twin^d^	0 (0)	0 (0)		–
2nd twin^e^	0 (0)	0 (0)		
CPAP (%)				
1st twin^d^	1 (4.8)	2 (1.8)		0.411
2nd twin^e^	2 (9.5)	8 (7.3)		0.664
Blood loss at delivery^f^ (ml)	500 (200–2500)	400 (200–3000)		0.064
Blood loss > 1 l (%)	6 (27.3)	21 (19.1)		0.393

Medians, ranges and percentages were estimated

^a^: 4 (DC/DA) unknown cases which are not taken into account

^b^: 5 (DC/DA) unknown cases which are not taken into account

^c^: 2 (DC/DA) unknown cases which are not taken into account

^d^: 1 (MC/DA) unknown case which is not taken into account

^e^: 1 (MC/DA) and 1 (DC/DA) unknown cases which are not taken into account

^f^: 3 (DC/DA) unknown cases which are not taken into account

In median, pH-values after vaginal birth did not differ significantly between MC-DA and DC-DA neonates (Table 2). Acidosis was found in 13.6% of the first and second MC-DA twins born vaginally, whereas 10.2% of first and 34.3% of second DC-DA twins were affected. There was no significant difference in acidosis rates for any twin after vaginal birth between MC-DA and DC-DA twins (Table 2).

Median 5-min Apgar score was nine in all compared groups. Apgar scores < 7 at 5 min postpartum were more often found in MC-DA twins (4.5% each) than in DC-DA twins (0.9 and 1.8%), but this was not statistically significant (Table 2). MC-DA and DC-DA twins’ median birth weights were similar for both first and second twins and did not differ significantly. Admission to NICU was necessary in 4.8 and 1.8% of the first twins of MC-DA and DC-DA births respectively and in 9.5 and 7.3% of the second twins without being statistically significant (Table 2). All children admitted to NICU required breathing support in the form of CPAP but intubation was required neither in vaginally born MC-DA nor in DC-DA twins. One child died during the 11-year period the deceased child was a DC-DA twin and died due to pulmonary hypoplasia.

Maternal outcome regarding postpartum blood loss was not statistically significant between the comparison groups (Table 2). Blood loss after MC-DA vaginal birth was in median 100 ml higher compared to DC-DA births (500 ml versus 400 ml) and postpartum haemorrhage > 1000 ml occurred more often after MC-DA births (27.3% versus 19.1%).

## Discussion

The optimal mode of birth for twin pregnancies remains a controversial topic and much research has been carried out, mainly regarding neonatal outcome. At the same time, rates of caesarean birth remain high. The CS rates in Germany have increased over the last 30 years and in 2019, 29.6% of all hospital births were caesareans [22]. The aim of this study was to analyse local data in order to gain an overview of neonatal outcomes and maternal complication rates of MC-DA versus DC-DA twins with planned vaginal births.

Of the 638 twin pregnancies found in the database between 2004 and 2014 which met the inclusion criteria, 47.3% were planned vaginal births and 52.7% caesarean, comparable the findings of Dathan-Stumpf et al. [23]. Similar to the results of Hoffmann et al. vaginal birth was planned for more MC-DA pregnancies than DC-DA pregnancies [15]. Other studies reported higher planned vaginal birth rates (72.9%) as well as lower rates (46.9%) [19, 20]. However, planned vaginal delivery remained higher in DC-DA pregnancies than in MC-DA pregnancies, which is in contrast to our findings [15, 19, 20]. The high total caesarean birth rate in this study is due to the high rate of planned caesareans for both MC-DA and DC-DA twins. In this study, sucessful vaginal delivery was less frequent for MC-DA (44.0%) and DC-DA (43.7%) pregnancies than in the investigations of Garabedian et al. and Schmitz et al. (52.6 and 54.7%; 81.7 and 79.6% respectively) but similar to the findings of Hoffmann et al. and Sau et al. (39.0 and 48.0%; 43.4 and 46.4% respectively) [15, 18–20]. The discrepancies between the various studies can be explained by differences in clinical guidelines and the size of population studied. German guidelines recommed delivery between 36 + 0 and 37 + 0 weeks for MC-DA twins and between 37 + 0 and 38 + 0 weeks’ gestation for DC-DA twins [24]. Garabedian et al. offered trials of labour in twin pregnancies with the first twin in breech position and in context of no CS on maternal request [20]. Schmitz et al. extracted their data from a large collective of 176 maternity units in France [19]. In the studied collective, total caesarean rates but also total vaginal birth rates were higher in MC-DA pregnancies as a result of a higher proportion of combined vaginal and caesarean births for DC-DA pregnancies.

Demographic differences between the subsamples in our study were found to be statistically non-significant, which is consistent with similar studies [16, 18, 20, 23]. In median, women planning a vaginal birth for both MC-DA and DC-DA twins were primiparae, which matches the findings of Garabedian et al. who also found no significant difference in parity between monochorionic and dichorionic twin pregnancies [20]. Pre-pregnancy BMI was higher in women with DC-DA twins who achieved vaginal birth but without reaching statistical significance. Garabedian et al. observed a smaller BMI for monochorionic pregnancies whereas Schmitz et al. presented a higher BMI for women with monochorionic pregnancies, but both differences were not significant [19, 20]. Most studies have sought an association between pre-pregnancy BMI and mode of birth for twins. Our analysis showed that women pregnant with MC-DA twins had a smaller BMI before delivery than DC-DA pregnancies. The association between BMI at birth for twins merits further investigations.

Gestational age at birth did not differ significantly between MC-DA and DC-DA twins in our analysis. Other studies have reported slightly lower gestational ages at birth for both monochorionic and dichorionic twins but these studies included pregnancies ≥24 or rather ≥28 weeks of gestation [18, 20].

Regarding neonatal ouctomes, our analysis showed the median Apgar at 5 min postpartum to be nine for MC-DA and DC-DA twins but Apgar scores < 7 were 5-times as likely for first and 2.5-times as likely for second MC-DA twins than for DC-DA twins after vaginal birth. The risk of an Apgar score < 7 in any of the twins was 1.7-times higher for monochorionic twins. These findings are in accordance with other studies which reported similar percentages of Apgar scores < 7 for mono- and dichorionic twin pregnancies [20, 23]. Sau et al. presented a significant difference with Apgar scores < 7 in 19.0% for MC-DA and in 5.0% for DC-DA twin pregnancies with vaginal birth [18].

There was no significant difference in median pH-values between vaginally born MC-DA and DC-DA twins and values were always in ther normal range according to the classification by Saling [21]. Only second twins of DC-DA pregnancies had a median pH-value that was preacidotic. Odds ratio for acidosis was 3.3-times more likely in vaginally born second DC-DA twins than in their comparison group. The risk of acidosis in any of the twins was 2-times more likely for dichorionic twins. Other studies also reported no significant differences in acidosis and pH-values between vaginally born monochorionic and dichorionic twins, but the percentages of acidosis differed between the studies which can be explained by the inclusion of lower gestational ages [18, 20, 23]. Our study found no significant difference in birth weights between the compared samples, while Schmitz et al. and Garabedian et al. found significant differences with lower birth weights for both twins, or rather the first monochorionic twin in comparison to dichorionic twins [19, 20]. Rates of admission to NICU in our analysis were similar to the findings of Dathan-Stumpf et al. and Garabedian et al. who showed slightly higher but non-significant rates for MC twins; second twins were also more often affected regardless of chorioncity [18, 20, 23]. Our research also revealed that breathing support was only required as CPAP; no intubation was needed by any of the twins. This confirms other findings which showed no significance, although they presented higher intubation rates for vaginally delivered MC-DA twins than for DC-DA twins [18, 19, 23]. One vaginally delivered DC-DA twin died in the 11-year period due to prenatally diagnosed pulmonary hypoplasia. Hoffmann et al. showed no neonatal deaths and Schmitz et al. also had one neonatal death in their investigation [15, 19].

Maternal outcome regarding postpartum blood loss showed no significant differences between the comparison groups which is supported by the results of Garabedian et al. [20].

In summary, the findings of our study were consistent with those of other researchers, who also found no significant differences in neonatal outcomes after vaginal birth when comparing MC-DA and DC-DA twins.

Both Garabedian et al. and Schmitz et al. showed that the outcomes for monochorionic and dichorionic twins are similar and vaginal birth can be offered to women regardless of chorionicty [19, 20]. Sau et al. also concluded that vaginal birth is safe for monochorionic twins when taking obstetric criteria into account and that transient neonatal outcomes were even worse after CS than after sucessful vaginal birth, which is also supported by the results of Dathan-Stumpf et al. [18, 23]. In contrast, Hoffmann et al. demonstrated a higher risk of adverse outcome after planned vaginal birth than after planned CS for dichorionic twins but not for monocchorionic twins [15]. While vaginal birth can be an option for mothers of both MC-DA and DC-DA twins, our research found that more than half of planned vaginal births resulted in a caesarean birth. The greater risk for mothers undergoing an unplanned CS must be kept in mind when counselling women on the mode of birth with twin pregnancies. The literature reports higher risks of short-term complications in mothers undergoing unplanned secondary CS compared to planned primary CS and vaginal delivery mainly due to higher anaesthesia- and surgery-related morbidity [24–26]. The results of our research, however, confirm that vaginal birth for monochorionic twins ≥36 weeks of gestation is just as safe as for women with DC-DA pregnancies.

### Strengths and limitations

This retrospective study in a university hospital setting has the advantage of a large sample size of twin pregnancies and covers a period of 11 years. However, the relatively small number of MC-DA twins is a reason why the differences we have found are not statistically significant. The analysed data are from 2004 to 2014 and are accordingly not entirely current, but they are very consitent and show good comparability with similar studies. The retrospective study design also has to be considered when comparing our results with other findings, as well as the fact that a level one perinatal hospital and fertility centre serves a high-risk population which can also affect results.

## Conclusion

We found no significant differences in neonatal or maternal outcomes after vaginal birth when comparing MC-DA and DC-DA twin pregnancies. DC-DA twins more often had acidosis whereas MC-DA twins were more often affected by Apgar scores < seven. Vaginal birth can be an option for twin pregnancies regardless of chorionicity. It must be kept in mind, however, that more than half of planned vaginal births resulted in CS, raising the risk of adverese maternal outcomes. The optimal mode of birth for twins has to be decided individually for every twin.

## Data Availability

The datasets generated and/or analysed during the current study are not publicly available due to the fact that the data falls under medical confidentiality but are available from the corresponding author on reasonable request.

## Abbreviations

BMI: Body mass index
CI: confidence interval
CS: Caesarean section
DC-DA: Dichorionic-diamniotic
NICU: Neonatal intensive care unit
MC-DA: Monochorionic-diamniotic
OR: Odds ratio
UFK: Department of Women’s Health, University Hospital of Tuebingen
